# Association between attention-deficit/hyperactivity disorders and intestinal disorders: A systematic review and Meta-analysis

**DOI:** 10.1038/s41598-025-04303-x

**Published:** 2025-06-02

**Authors:** Rita W. Y. Ng, Zigui Chen, Liuyue Yang, Oscar W. H. Wong, Agnes S.Y. Leung, K. W. Tsui, Natalie M.W. Kwok, Lesley H.Y. Tang, Peter M.H. Cheung, Paul K. S. Chan, Margaret Ip

**Affiliations:** 1 Department of Microbiology, Faculty of Medicine, The Chinese University of Hong Kong, Prince of Wales Hospital, Shatin, Hong Kong (SAR), N/A China; 2 Department of Psychiatry, Faculty of Medicine, The Chinese University of Hong Kong, Prince of Wales Hospital, Shatin, Hong Kong (SAR), N/A China; 3 Department of Paediatrics, Faculty of Medicine, The Chinese University of Hong Kong, Prince of Wales Hospital, Shatin, Hong Kong (SAR), N/A China; 4 Department of Paediatrics and Adolescent Medicine, Alice Ho Miu Ling Nethersole Hospital, Hospital Authority, Hong Kong (SAR), China; 5 Faculty of Medicine, The Chinese University of Hong Kong, Sha Tin, Hong Kong (SAR), China; 6 Shenzhen Research Institute, The Chinese University of Hong Kong, Shenzhen, 518057 China

**Keywords:** Attention-deficit hyperactivity disorder (ADHD), Intestinal disorders, Meta-analysis, Microbiology, Gastroenterology

## Abstract

Patients with attention-deficit hyperactivity disorder (ADHD) reported significantly more constipation and flatulence than healthy controls. An altered gut microbiome can be associated with gastrointestinal symptoms. However, comprehensive information about associated risk of intestinal disorders and ADHD remains limited. A systematic review of the literature was therefore conducted to investigate the association between ADHD and different types of intestinal disorders. A total of 11 studies with 3,851,163 unique individuals, including 175 806 individuals with ADHD and 3 675 357 individuals without ADHD were included. The pooled OR of intestinal disorders for individuals with ADHD was 1.25 (95%CI, 0.75–2.07). A significant positive association was found between ADHD and irritable bowel syndrome (IBS) (OR 1.63 [95% CI 1.45–1.83]). Studies conducted in Eastern Mediterranean Region yielded a summary OR estimate that was higher than summary OR estimates in studies conducted in Region of the Americas, European Region and Western Pacific Region (3.03 [1.53–5.99] vs. 2.20 [1.05–4.63], 1.04 [0.44–2.41], 0.68 [0.25–1.87]), with p value 0.053, indicating a trend towards significance. High heterogeneity was observed. Our study supports association between ADHD and increased risk of IBS. Our study suggests an altered gut microbiome is the potential link that bridges gap between ADHD and intestinal disorder.

**Supplementary Information:**

The online version contains supplementary material available at 10.1038/s41598-025-04303-x.

## Introduction

Attention-deficit hyperactivity disorder (ADHD) is a chronic neurodevelopmental disorder characterised by inappropriate levels of inattention, hyperactivity and impulsiveness, which usually begins its course from childhood and persists through adulthood^1^. ADHD is one of the most prevalent neuropsychiatric childhood disorders with prevalence ranging from 3.4 to 14%^2^. The prevalence of symptomatic adult ADHD was 6.76%, translating to 366.33 million affected adults in 2020 globally^3^. ADHD has been linked to a broad range of adverse consequences for affected individuals, including increased risk of smoking^4^, substance-related disorders^5^, sexual risk-taking behaviour^6^, greater driving risk^7^ and suicidal behaviour^8^. The chronic debilitating disease poses a significant economic burden on families and society^9^.

Clinically, ADHD is characterized by specific behavioural traits with inattention and hyperactivity. In addition, psychological comorbidity with ADHD is substantial. ADHD is known to co-occur with various neuropsychiatric disorders, including autism spectrum disorder, learning disorders, tic disorders, depressive disorder, bipolar disorder, anxiety disorder, conduct disorder and oppositional defiant disorder^10^. Apart from neuropsychiatric disorders, ADHD has been associated with increased risk of medical conditions including obesity, sleep disorder and asthma^11^. These factors contribute to its classification as a significant public health concern^12^.

Intestinal disorders are a wide spectrum of diseases describing diseases originating from the intestine or affecting the normal function of digestion and absorption of the gastrointestinal (GI) tract^13^. The exact cause of the certain types of intestinal disorders are entirely unclear. Intestinal disorders can be classified into different types. Functional intestinal disorder is a spectrum of chronic gastrointestinal disorders characterized by symptoms or signs of abdominal pain, bloating, distention and bowel habit abnormalities, including irritable bowel syndrome (IBS)^14^. Insufficient evidence exists regarding the etiology of other inflammatory intestinal disorders including inflammatory bowel disease (IBD)^15^ and celiac disease^16^. There is increasing evidence suggesting the role of gut microbiota in developing intestinal disorders and neuropsychiatric disorders. Gut-brain axis^17^ refers to the network of connections involving multiple biological systems that allows bidirectional communication between gut bacteria and the brain. The healthy gut microbiome refers to the collection of bacteria, viruses, bacteriophages, protozoa and fungi residing in the human gut that confer health benefits^18^. Dysbiosis, in contrast, is the imbalanced state of gut microbial communities that can lead to dysregulation of bodily functions and various diseases^19^. An altered gut microbiome has been implicated in various neuropsychiatric disorders including Parkinson’s disease, multiple sclerosis, Alzheimer’s disease and autism spectrum disorders^20^. An altered gut microbiome can be associated with gastrointestinal symptoms, including constipation, diarrhoea and flatulence. Children with ADHD reported significantly more constipation and flatulence symptoms than the healthy controls^21^.

Given the abundance of studies trying to connect the gut-brain-axis with ADHD, an investigation into whether ADHD patients exhibit more gastrointestinal pathologies than the neurotypical population is the first step to identify dysbiosis in ADHD. The objective of this meta-analysis is to consolidate the available literature on the association between ADHD and intestinal disorders including IBS, IBD, celiac disease, constipation, dyspepsia, recurrent abdominal pain, Shigellosis, peptic ulcer, and fecal incontinence from different geographical regions in the East and West, and to shed light on future investigations and management of intestinal disorders in patients with ADHD.

## Method

This meta-analysis was developed per the Preferred Reporting Items for Systematic Reviews and Meta-Analyses^22^ reporting guideline. The study was registered in the PROSPERO International Prospective Register of Systematic Reviews (CRD42023486812).

### Study design

A systematic literature search of key databases, including EMBASE, Medline, Web of Science and APA PsycINFO, was conducted to identify relevant studies from their inception until 16 November 2023. Search terms were related to diarrhoea, bowel disease, bowel disorder, inflammatory bowel, Crohn’s disease, colitis, mucous, colon, irritable bowel, irritable bowel syndrome, functional gastrointestinal disorder, ulcerative colitis, constipation, colonic inertia, dyschezia, small intestinal bacterial overgrowth, ADHD, ADDH, behavior disorder, disruptive, attention deficit, hyperkinetic, hyperactivity, minimal brain dysfunction, and can be found in Appendix 1 in Supplement. Grey literature was searched via Google Scholar using the search terms and the reference list of included articles.

### Study selection

Studies included in the meta-analysis were control studies, nested case-control studies, cohort studies and cross-sectional studies. We included studies that met the following criteria^1^: including patients diagnosed with ADHD by medical professionals using standardized ADHD diagnosis criteria of all ages^2^; reporting the number of patients with intestinal disorders^3^; providing odds ratios (ORs) or relative risks (RRs) or hazard ratio (HR) with corresponding 95% CIs as measures of association or allowing for computation of these measures based on count data reported in the article; and^4^ published in English. We excluded studies that^1^ constituted narrative and/or systematic reviews, commentaries, editorials, conference abstracts or theses; and^2^ lacked a clear clinical definition of ADHD or intestinal disorder.

### Data extraction

After removing duplicates, titles and abstracts were screened, followed by full-text screening. Screening was completed by 2 independent reviewers (Kwok and Tang), and full text review was completed by 2 independent reviewers (Tang and Cheung) according to eligible criteria set by review team, with discrepancies resolved via discussion among the review authors. A data extraction template was developed, and the following information was extracted for each study by 2 independent reviewers (Cheung and Kwok): authors, year of publication, country, continent, study design, study type, age group, ADHD treatment, ADHD diagnosis criteria, data source of study, number of patients with ADHD, number of patients without ADHD, intestinal disorders with ADHD and intestinal disorders in patients without ADHD and type of intestinal disorder. Included studies were assessed for internal validity and bias risk using the critical appraisal tool, Joanna Briggs Institute (JBI) Appraisal Checklist for reporting prevalence data^23^. The JBI tool is found in Appendix 2 in Supplement. The research team decided that good-quality studies were required to score 70% or greater (score of > = 7 of 9), moderate-quality studies needed to score 50% to less than 70%(score of 5 or 6 of 9), and poor-quality studies scored less than 50% (score of < = 4 of 9). These quality assessment threshold scores have been used in past reviews^24^. Quality assessment was completed on all included studies by 2 independent reviewers (Yang and Ng). Any disputes relating to quality assessment between the reviewers were resolved by discussion with senior supervisor (Ip).

### Statistical analysis

The primary outcome measure was the OR of intestinal disorders among individuals with ADHD compared with those without and the secondary outcome measure was the OR of specific type of intestinal disorder. The included studies for data analysis^25–35^ were case control studies, cohort or cross-sectional studies. A random-effects meta-analysis was chosen for meta-analysis. Statistical heterogeneity between the studies was evaluated using univariate meta-regression and subgroup analysis for age group, study type, study design, World Health Organization (WHO) region, number of subjects, and ADHD diagnosis criteria with the *I*^*2*^ statistic and Cochran Q test. Heterogeneity was considered an issue if the *I*^*2*^ statistic was greater than 40% and/or the Q statistic was significant at 2-sided *P* = 0.01^36^. Leave-one-out method was used in the sensitivity analysis. The Egger’s test was used to assess publication bias. Package ‘meta’ and ‘metafor’ in the R environment was used throughout the data analysis.

## Results

### Study selection

**Fig. 1:**
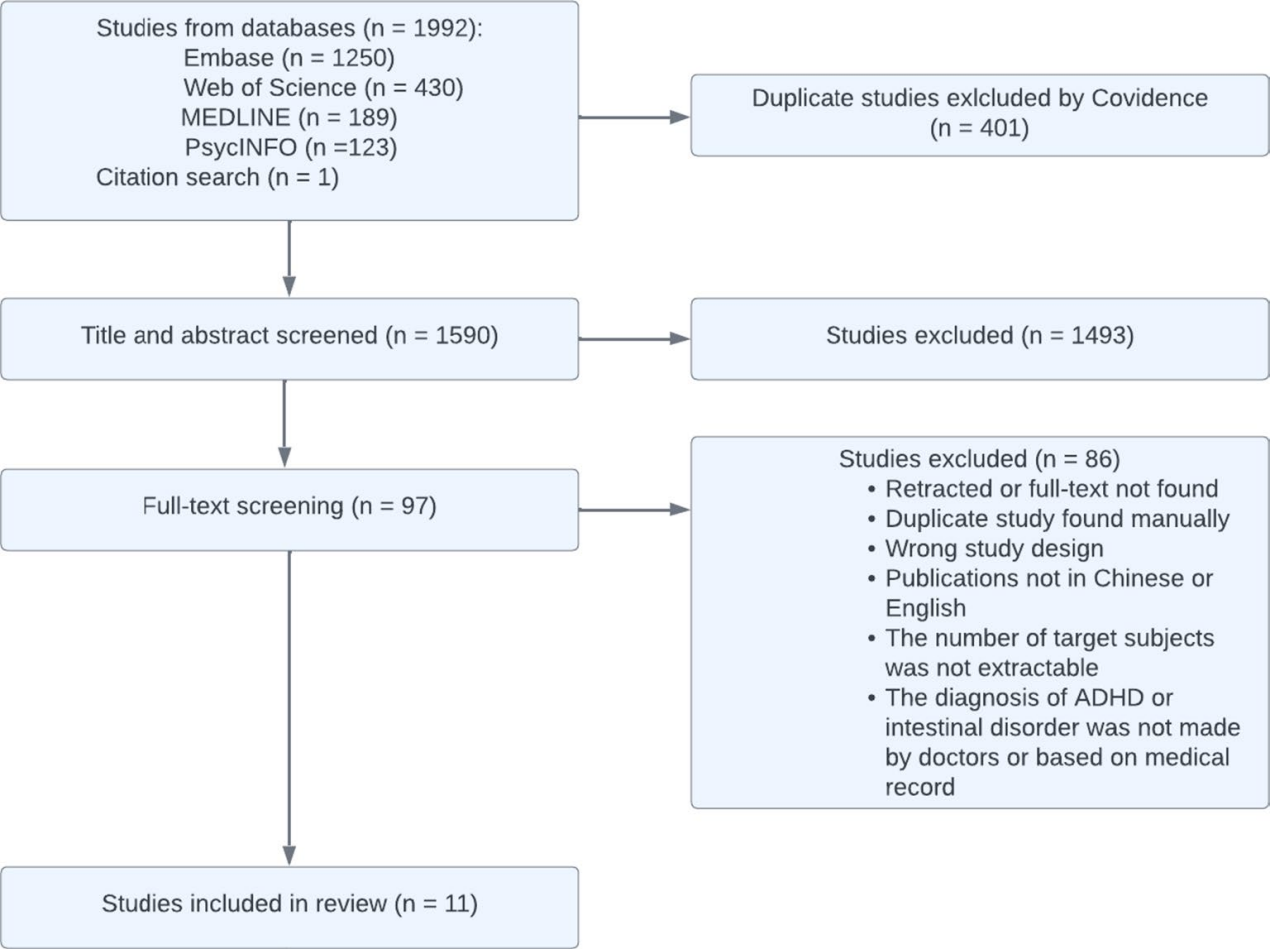
Preferred reporting items for systematic reviews and meta-analyses (PRISMA) flowchart.

A total of 1992 studies were initially identified through a systematic search. Following a process of duplicate exclusion and abstract screening, 97 studies were considered potentially relevant and subjected to a thorough full-text review. Finally, 11 studies were included, encompassing a combined study population of 3,851,163 individuals, including 175 806 individuals with ADHD and 3 675 357 individuals without ADHD. The stepwise selection process is depicted in Fig. 1.

The publication timeline of the included studies varied, with 2 studies published in 2000 s, 5 studies published in 2010 s and 4 studies published in 2020 s. Geographically, the studies covered a wide range of regions, with 2 studies conducted in North America, 4 in Europe and 5 in Asia. Five cohort studies, five case-control studies and one cross-sectional study were included. The World Health Organization’s Ninth Revision, International Classification of Diseases (ICD-9) was used for ADHD diagnosis in 5 (45.5%) studies, while Diagnostic and Statistical Manual of Mental Disorders (DSM) was used in 3 (27.3%) studies, and both diagnostic criteria were used for diagnosis in 1 (9.1%) study. 9 (81.8%) studies were retrospective studies, while 2 (18.2%) studies were prospective studies. The detailed characteristics of each included study can be found in Table 1.

**Table 1:** Baseline characteristics for the 11 studies included in meta-analysis.

Study name	Country	Continent	Study design	Study type	Age group	ADHD treatment	ADHD diagnosis criteria/system	Data source of retrospective study	Total ADHD	Total non-ADHD	Intestinal disorders in ADHD	Intestinal disorders in non-ADHD	OR (95% CI)	Quality score
Chen et al., 2017	Taiwan	Asia	Case-control study	Retrospective	All ages	Unclear	ICD-9-CM^a, b^	Taiwan National Health Insurance Research Database (NHIRD)	8201	32,804	124	446	1.11 (0.91–1.36)	100%
Gungor et al., 2013	Turkey	Europe	Case-control study	Prospective	Children	Unclear	DSM^c^-IV	NA	362	390	4	3	1.44 (0.32–6.48)	100%
Hegvik et al., 2018	Norway	Europe	Cross-sectional study	Retrospective	All ages	Yes	Reimbursed ADHD medication (ATC N06BA)	Medical Birth Registry of Norway (MBRN) and Norwegian Prescription Database (NorPD)	63,721	2,436,397	342	12,242	1.07 (0.96–1.19)	100%
Hodgkins et al., 2011	US	North America	Case-control study	Retrospective	Adult	Yes	ICD-9-CM (314.0, 314.00, 314.01)	MarketScan Commercial Claims and Encounters (commercial) and the Health and Productivity Management (HPM) databases	31,752	95,256	286	572	1.50 (1.30–1.73)	89%
Holmberg et al., 2006	Stockholm	Europe	Cohort study	Prospective	Children	Unclear	DSM-IV	NA	29	420	12	83	2.87 (1.32–6.23)	60%
Hu et al., 2021	Taiwan	Asia	Cohort study	Retrospective	Adult	Yes	ICD-9 codes (314)	Taiwan National Health Insurance Research Database (NHIRD)	798	2394	19	139	0.40 (0.24–0.64)	90%
Kedem et al., 2020	Israel	Asia	Cohort study	Retrospective	Adult	Yes	DSM and ICD-9 (314.0, 314.00, or 314.01)	Israel Defense Forces	33,380	355,652	2794	19,703	1.56 (1.49–1.62)	90%
Mckeown et al., 2013	US	North America	Cohort study	Retrospective	Children	Yes	ICD-9-CM (314.00, 314.01, 314.1, 314.2, 314.8, or 314.9	TRICARE Management Activity military health system (MHS) database.	32,773	710,166	1648	11,530	3.21 (3.04–3.38)	90%
Merzon et al., 2021	Israel	Asia	Cohort study	Retrospective	Children	Unclear	ICD-9 codes (314.00–314.9)	Health Maintenance Organization database	4601	41,541	556	4033	1.28 (1.16–1.40)	100%
Van Den Heuvel et al., 2007	The Netherlands	Europe	Case-control study	Retrospective	Children	Unclear	DSM-IV	Hospital record	146	133	2	23	0.07 (0.02–0.29)	78%
Zafari et al., 2021	Iran	Asia	Case-control study	Retrospective	Children	Unclear	Persian version of Conners’ Parent Rating Scale (short form)	NA	43	204	27	73	3.03 (1.53–5.99)	89%

Abbreviations: OR, odds ratio, NA, not available.

^a^ICD-9:World Health Organization’s Ninth Revision, International Classification of Diseases.

^b^CM: Clinical modification.

^c^DSM: Diagnostic and Statistical Manual of Mental Disorders.

### Quality assessment of the included studies

The JBI quality checklist (https://jbi.global/critical-appraisal-tools) determined that most the studies were of good quality (10 [91%]). One study (9%) was of moderate quality. No studies were excluded from the main meta-analysis based on the JBI score. The quality assessment score for each study is in Table 1.

### ADHD and intestinal disorders

**Fig. 2:**
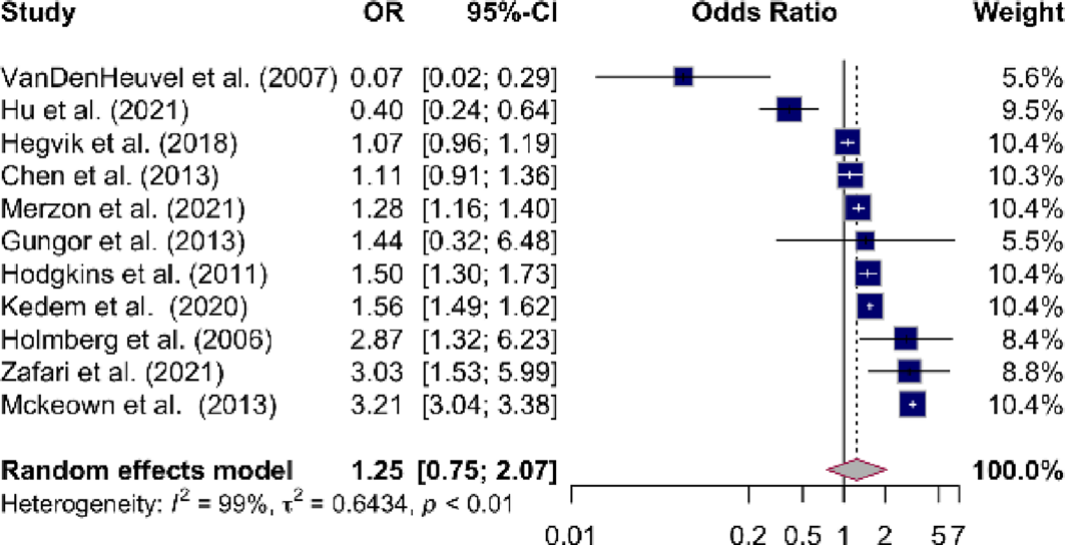
Forrest plot showing odds ratios (OR) for all types of intestinal disorders in ADHD. The size of the box representing the point estimate for each study in the forest plot is proportional to the contribution of that study’s weight estimate to the summary estimate. The diamond represents the pooled odds ratio (OR); the lateral tips of the diamond represent the associated 95%CIs.

The association between ADHD and intestinal disorders was investigated in 11.

studies. The summary estimate of ADHD as a risk factor for intestinal disorders was OR of 1.25 (95%CI, 0.75–2.07), with heterogeneity of *I*^*2*^ = 99% (*P* < 0.01). Figure 2 showed the overall risk estimates of association between ADHD and all types of intestinal disorders reported in the included studies, including IBS, IBD subtype, celiac disease, constipation, dyspepsia, recurrent abdominal pain, Shigellosis, peptic ulcer, and fecal incontinence.

### Association between ADHD and all types of intestinal disorders

**Fig. 3:**
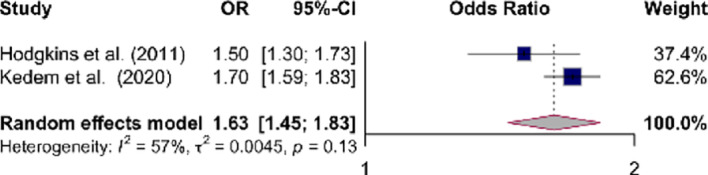
showed a significant positive association was found between ADHD and irritable bowel syndrome (IBS) (OR 1.63 [95% CI 1.45–1.83]) (I2 = 57%, Tau2 = 0.0045 *p* = 0.13). No association was found in case of IBD subtype (Crohn’s disease and ulcerative colitis), celiac disease and constipation.

Figure 3: Forrest plot showing odds ratios (OR) for irritable bowel syndrome in ADHD. The size of the box representing the point estimate for each study in the forest plot is proportional to the contribution of that study’s weight estimate to the summary estimate. The diamond represents the pooled odds ratio (OR); the lateral tips of the diamond represent the associated 95%CIs.

### Meta-regression and subgroup analysis

Meta-regression and subgroup analysis, stratified by various study-level characteristics, were conducted to explore sources of heterogeneity. In univariate meta-regression, none of the study-level characteristics were found to be significantly associated with the outcome, although WHO regions had the largest contribution (R² = 1.17%). Similarly, in the subgroup analysis, studies conducted in Eastern Mediterranean Region yielded a summary OR estimate that was higher than the summary OR estimates in studies conducted in Region of the Americas, European Region and Western Pacific Region (3.03 [1.53–5.99] vs. 2.20 [1.05–4.63], 1.04 [0.44–2.41], 0.68 [0.25–1.87]), with p value 0.053, indicating a trend towards significance (Appendix 3 in Supplement). No statistically significant differences in OR estimates were found between subgroups of studies stratified by age group (adults, children, or subjects of all ages), study type, study design (case-control study, cross-sectional study, or cohort study) and number of subjects (less than 1000 versus equal or more than 1000).

### Sensitivity analysis

Appendix 4 in Supplement showed Leave-one-out analysis. After excluding 1 potential outlier (Van den Heuvel et al.) studying constipation among children, a case-control study with a relatively small sample size and the use of outpatient control (instead of community control), the pooled odds ratio (OR 1.47 [95% CI 1.00–2.16] revealed a statistically significant positive association between ADHD and intestinal disorders.

### Publication Bias

**Fig. 4:**
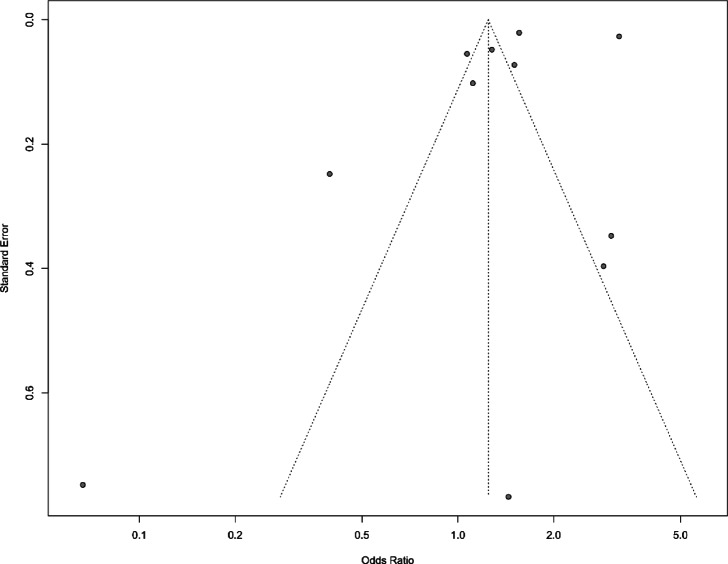
Publication bias regarding ADHD and risk of intestinal disorders with study-specific information. The middle line indicates the overall effect of the meta-analysis, while the 2 lines on either side represent the 95%CIs.

Figure 4 shows a funnel plot illustrating the potential publication bias. The studies are distributed around the pooled effect size (indicated by the vertical line at the center), both within and outside the funnel’s contours. This suggests a reduced likelihood of significant bias in smaller studies, which are more susceptible to yielding nonsignificant results and therefore potentially going unnoticed. Egger’s test does not provide strong evidence for the presence of funnel plot asymmetry and publication bias in the meta-analysis (p-value = 0.38).

## Discussion

This systematic review and meta-analysis comprehensively summarized the available literature and revealed an association between ADHD and IBS. Notably, in subgroup analysis, there was a trend that studies conducted in Eastern Mediterranean Region yielded a summary OR estimate of intestinal disorders that was higher than those in Region of the Americas, European Region and Western Pacific Region (*p* = 0.053).

ADHD has been associated with increased risk for intestinal disorders^21^. Compared with previous studies, the novelty of our study lies in providing the pooled estimates of the published studies with a wide range of international population using meta-analysis. Limited research existed to study the association between intestinal disorders with ADHD with inconsistent findings. Some studies reported a higher rate of encopresis^37^, stomach bloating and cramps^38^ in children of ADHD compared with those without ADHD. Other study showed no difference of gastrointestinal problems in ADHD and non-ADHD twins^39^. In our study, more detailed and specific data focusing on ADHD and different types of intestinal disorders with clinical diagnostic criteria determined by clinical professionals instead of patients’ symptoms would be useful for a broad range of physicians as well as parents.

Our results showed a trend towards significance for the subgroup difference of the risk of intestinal disorders in ADHD in different WHO regions, with OR estimate of Eastern Mediterranean Region higher than those in Americas, European Region and Western Pacific Region. Differences in ADHD prevalence in different geographical regions^40^ may be due to differences in study methodologies, including sample size, response rate, information sources from parents or teachers, cultural factors and clinical diagnosis criteria of ADHD. Similar ADHD patient populations from North American and non-North America were identified with the same diagnostic assessment, yielding a uniform disease prevalence with the use of standardized diagnostic criteria^41^. This study highlights the importance of standardized ADHD diagnostic criteria when researchers compare data from different regions.

Our study showed a positive association between ADHD and IBS, with odds of IBS in ADHD patients 1.63 times greater than that in controls. The etiology of IBS was poorly understood as it is often multifactorial. It has been postulated that IBS pathogenesis involves altered or overgrowth of gut microbiome, hypersensitivity to food, post infection reactivity or inflammation, and altered intestinal motility^42^. Specifically, IBS patients had significantly lower alpha diversity and enrichment of Gram-negative bacteria with reduction in pathways associated short-chain fatty acid, differentiating microbial features in healthy and IBS subtypes^43^. Our results suggest that gut microbiome may explain the link between ADHD and IBS. Our finding of the positive association between ADHD and IBS suggests that clinicians should be aware of gastrointestinal symptoms in children and adults with ADHD. Gastrointestinal symptoms were independently associated with aberrant behaviour checklist in patients with autism spectrum disorder (ASD) and ADHD^44^. Screening of IBS may be considered during assessment of patients with ADHD, especially those presenting with abdominal discomfort or other gastrointestinal complaints. Appropriate assessment of intestinal disorders in patients with ADHD may help diagnosis and early management. This could improve quality of life in patients with neurodevelopmental disorders and their families.

IBS considerably affects quality of life in affected patients. Its presence in ADHD patients may further complicate the management of ADHD. Treatment of gastrointestinal symptoms due to IBS in ADHD patients could improve overall functioning of ADHD patients. On the other hand, methylphenidate, the first line treatment for ADHD, increases the risks of abdominal pain in ADHD patients^45^. There is a need for clinicians to adopt a more proactive approach in the assessment gastrointestinal symptoms in patients with ADHD. Although patients with ADHD experience increased direct health care costs^46^, there is lack of evidence of prediction of later development of more severe gastrointestinal conditions in childhood ADHD patients suffering from IBS or other gastrointestinal disorders. More studies are needed on the long-term clinical outcome of ADHD patients.

As a neurodevelopmental disorder, ADHD still remains a chronic disease with largely unexplained etiology. There is evidence of strong genetic component of common variants in the polygenic architecture of ADHD with 22% of Single Nucleotide Polymorphism (SNP)-based heritability^47^. Although considered a highly familial disorder, ADHD heritability estimates of 60–80% highlight a potential role of environmental factors in the susceptibility of this disorder^48^. ADHD has been associated with an increased oxidative stress and neuroinflammation^49^. Recent studies have highlighted the potential influence of gut microbiota on health and disease. Gut microbial therapy by administration of probiotics, synbiotics and prebiotics may alleviate the medical condition in specific disorders, for example reduction of C-reactive protein^50^, low-density lipoprotein (LDL)^51^, alanine aminotransferase and aspartate aminotransferase^52^ and insulin resistance^53^ among non-alcoholic fatty liver disease (NAFLD). Gut microbiome is recognized as a key player in the pathogenesis of ADHD in gut-brain axis^54,55^, where the neural, endocrine and immunological pathways are involved this communication interplay^56^. Alterations in gut microbiome in ADHD were reported in previous studies, with ADHD group showing higher level of Dialister and Megamonas and lower abundance of Anaerotaenia and Gracilibacter at the genus level^57^, and Bacteroides species correlating with levels of hyperactivity and impulsivity^58^. Microorganisms influence the brain through their ability to produce and modify metabolic, immunological and neurochemical factors in the gut that ultimately impact on the central nervous system^59^. In turn, brain activity also impacts the gut microbiota composition^60^. The gut microbiota influences gut barrier and produce neurotransmitters, amino acids and microbial metabolites^61^. This dynamic bidirectional communication between the gut microbiota and the central nervous system influences brain function, cognition and behavior. Future studies are needed to elucidate the role of the gut microbiota in ADHD and the associated comorbid medical conditions including intestinal disorders.

Microbiota-targeted interventions, including mixture of probiotic and prebiotic, was shown to increase propionic acid levels in children with ADHD, contributing to lowering of higher-than-normal proinflammatory intercellular adhesion molecule 1 levels in a randomized controlled trial^62^. Higher therapeutic efficacy was associated with the usage of probiotics as an adjunct to methylphenidate in the treatment of ADHD^63^. Future studies are warranted to systemically investigate the role of microbiota-targeted interventions in the treatment of ADHD.

Alterations of gut microbiome is associated with pathogenesis of various intestinal disorders. Gut dysbiosis in Crohn’s disease and ulcerative colitis is characterized by reduced abundance of the phylum Firmicutes and increase in the phylum Proteobacteria^64^. Depletion of butyrate-producing bacteria results in a metabolic reorientation of surface colonocytes towards anaerobic glycolysis and an increase of oxygen diffusion into the lumen leading to luminal aerobe and/or facultative anaerobe expansion^65^. On the other hand, environmental stimuli may play a role in Celiac disease, another autoimmune disease with unknown etiology. Alterations in the gut microbiota, functional pathways and metabolome were identified in a prospective study of infants who developed Celiac disease. Increased abundances of several microbial species, including Dialister invisus and Parabacteroides species were detected before onset of Celiac disease, which were previously linked to autoimmune conditions^66^.

## Limitations

This meta-analysis has several limitations. First, nearly half of studies were from Asia, which may lead to bias and lack of certainty in generalizing the results to other regions. Second, heterogeneities between examined studies warrant attention. The study population differed to a certain degree, for example, some studies focused on specific individuals, 6 studies included children, 3 studies included adults and 2 included participants of all ages. These discrepancies in the findings may be due to differences in study design, study population, diagnostic criteria for ADHD and GI diseases. ICD has a stricter clinical definition for ADHD while DSM is more lenient which depends on operational criteria requiring combination of criteria. Third, this meta-analysis has a small sample size including data from 11 studies. Fourth, the small number of studies included in some of the groups may have biased some subgroup analysis results. Fifth, the finding of WHO regional differences for the association of ADHD and intestinal disorders indicating a trend towards significance may be due to a small sample size, but this noteworthy difference warrants further investigation. Sixth, majority of the studies (*n* = 9) included in this meta-analysis were retrospective studies, which were prone to recall bias and incomplete data collection. This might result in overestimation or underestimation of the true relationship between ADHD and risk of intestinal disorders.

## Conclusions

To conclude, our study showed no significant association between ADHD and all types of intestinal disorders, but it demonstrated an increased incidence of irritable bowel syndrome among ADHD patients. Further studies are needed for systemic investigation of the role of intestinal microbiota in ADHD. An altered gut microbiome and its inflammatory effect is the potential link that bridges the gap between ADHD and intestinal disorder. The exact detailed mechanism of dysbiosis and its effect on inflammatory dysregulation is yet to be elucidated in future studies. Hence, it would be important to conduct both basic science and clinical studies to investigate any possible changes in neurotransmitters, neurometabolism, and neuroanatomy that may be caused by dysbiosis. More studies are warranted to confirm a significant relationship between ADHD and intestinal disorders.

## Electronic supplementary material

Below is the link to the electronic supplementary material.

Supplementary Material 1

## Data Availability

The dataset analyzed in this study is available from the corresponding author upon reasonable request.
